# Circular RNAs as diagnostic biomarkers in type 2 diabetes mellitus: implications in metabolic dysfunction and immune-inflammatory crosstalk

**DOI:** 10.3389/fimmu.2026.1815722

**Published:** 2026-05-15

**Authors:** Zhaoxia Liu, Hui Wu, Qiaoshuang Pu, Xiongtao Yang, Da Li, Shipin Feng, Yan Wang

**Affiliations:** 1 General Medicine, Sichuan Clinical Research Center for Cancer, Sichuan Cancer Hospital & Institute, Sichuan Cancer Center, University of Electronic Science and Technology of China, Chengdu, China; 2 Department of Pediatric Nephrology, Chengdu Women’s and Children’s Central Hospital, School of Medicine, University of Electronic Science and Technology of China, Chengdu, China

**Keywords:** circular RNAs, competing endogenous RNA (ceRNA), diagnostic biomarkers, metabolic dysfunction, type 2 diabetes mellitus

## Abstract

Type 2 diabetes mellitus (T2DM) is a metabolically heterogeneous disease for which current approaches to early diagnosis and risk stratification remain limited. Circular RNAs (circRNAs) have emerged as promising molecular candidates because of their stability, disease relevance, and regulatory versatility. Circular RNAs (circRNAs) have emerged as a promising class of non-coding RNAs that exhibit unique advantages, such as high stability, disease specificity, and the ability to act as regulators in competing endogenous RNA (ceRNA) networks. This review summarizes the current understanding of circRNAs in T2DM, highlighting their potential as diagnostic biomarkers and their roles in metabolic dysfunction. We explore how circRNAs contribute to key pathogenic processes in T2DM, including insulin resistance, glucose uptake, β-cell survival, inflammation, and vascular dysfunction. Additionally, we discuss the challenges in circRNA research, including technical biases, cohort heterogeneity, and the need for rigorous mechanistic validation. The review concludes by outlining future directions for circRNA-based research, including multicenter prospective studies, single-cell and spatial transcriptomic integration, and the development of circRNA-based companion diagnostics for precision medicine in T2DM.

## Highlights

- Emerging role of circRNAs as diagnostic biomarkers in T2DM: CircRNAs are identified as stable, disease-specific molecules that can distinguish between various glycemic states and predict complications such as cardiovascular and vascular diseases.
- CircRNA-mediated ceRNA networks in metabolic regulation: CircRNAs regulate insulin resistance, glucose uptake, and β-cell function via ceRNA interactions, providing mechanistic insights into the progression of T2DM.
- Challenges and future directions: The review discusses current limitations in circRNA research, including sample size and cohort heterogeneity, and highlights future avenues for integrating circRNAs into precision medicine, with a focus on multicenter studies and advanced transcriptomic technologies.

## 1 Introduction

Type 2 diabetes mellitus (T2DM) represents one of the most prevalent and complex metabolic disorders worldwide and poses an escalating burden on global health systems (1–4). Characterized by chronic hyperglycemia resulting from insulin resistance and progressive pancreatic β-cell dysfunction, T2DM is increasingly recognized as a heterogeneous disease encompassing diverse genetic, metabolic, inflammatory, and environmental determinants (5–10). Despite substantial advances in understanding its pathophysiology, current clinical management of T2DM remains challenged by delayed diagnosis, incomplete risk stratification, and limited tools for predicting disease progression and complications.

A major clinical challenge in T2DM lies in the identification of reliable biomarkers for early detection and disease monitoring. Traditional diagnostic indicators, such as fasting plasma glucose, oral glucose tolerance testing, and glycated hemoglobin (HbA1c), primarily reflect glycemic status rather than underlying molecular alterations. These markers are often insufficient to capture early metabolic dysregulation, inter-individual heterogeneity, or the risk of developing vascular and metabolic complications. Moreover, many patients exhibit substantial metabolic impairment before meeting diagnostic thresholds, underscoring the urgent need for sensitive, non-invasive biomarkers that reflect disease biology rather than downstream metabolic consequences.

In recent years, non-coding RNAs (ncRNAs) have emerged as critical regulators of metabolic homeostasis and disease progression. MicroRNAs (miRNAs) and long non-coding RNAs (lncRNAs) have been extensively investigated in the context of insulin signaling, β-cell function, inflammation, and lipid metabolism. However, translational application of many ncRNA candidates has been hindered by limited stability, inconsistent detectability in circulation, and context-dependent expression patterns (11–16). These limitations have prompted increasing interest in circular RNAs (circRNAs), a distinct class of endogenous ncRNAs generated through back-splicing events that covalently link their 3′ and 5′ ends to form a closed loop structure. CircRNAs possess several properties that make them particularly attractive as diagnostic biomarkers and regulatory molecules in metabolic diseases. Owing to their circular configuration, circRNAs are highly resistant to exonuclease-mediated degradation, conferring remarkable stability in tissues, plasma, serum, and other body fluids. Accumulating evidence indicates that circRNAs exhibit cell-type, tissue-specific, and disease-specific expression patterns, suggesting that they may reflect distinct pathological states in T2DM. Importantly, circRNAs are abundantly detected in peripheral blood and extracellular vesicles, including exosomes, highlighting their potential as minimally invasive biomarkers for clinical application (17, 18).

Beyond their diagnostic value, circRNAs have emerged as active regulators of gene expression and metabolic signaling. Among their diverse functional modalities, the competing endogenous RNA (ceRNA) mechanism has attracted particular attention. Through acting as molecular sponges for miRNAs, circRNAs can modulate the availability of miRNAs to their target mRNAs, thereby shaping downstream transcriptional and translational programs (19). In T2DM, circRNA-mediated ceRNA networks have been implicated in key pathological processes, including insulin resistance, glucose uptake, β-cell dysfunction, chronic low-grade inflammation, lipid metabolism dysregulation, and vascular endothelial injury. High-throughput transcriptomic profiling and bioinformatic network analyses have further revealed that circRNAs occupy central nodes within complex regulatory circuits linking metabolic stress to cellular dysfunction.

Notably, emerging studies suggest that circRNAs may bridge molecular mechanisms with clinical phenotypes in T2DM. Specific circRNAs have been reported to discriminate between normoglycemia, impaired fasting glucose, and overt diabetes, while others correlate with disease duration, insulin resistance indices, or the presence of vascular complications. Moreover, circRNA expression patterns have been associated with metabolic comorbidities such as non-alcoholic fatty liver disease and cardiovascular disease, supporting their potential utility in disease stratification and risk prediction. The detection of circRNAs within circulating exosomes further expands their translational relevance, as exosome-encapsulated circRNAs may serve not only as biomarkers but also as mediators of intercellular communication in metabolic tissues. Despite rapid progress, the field of circRNAs in T2DM remains fragmented, with studies varying widely in experimental design, sample sources, analytical platforms, and functional validation strategies. A comprehensive synthesis of current evidence is therefore required to clarify the diagnostic potential of circRNAs, delineate both ceRNA-dependent and non-ceRNA regulatory mechanisms, and contextualize their roles in metabolic dysfunction. Such an integrative perspective is important because circRNA functions in T2DM are likely to be highly context-dependent, varying according to cell type, metabolic stress, and disease stage (20–22). Integrating these aspects is essential for advancing circRNAs from descriptive biomarkers toward mechanistically informed and clinically actionable targets.

In this review, we summarize emerging evidence on circular RNAs in type 2 diabetes mellitus, with a particular focus on their value as diagnostic biomarkers, their involvement in ceRNA regulatory networks, and their contributions to metabolic dysfunction. By integrating findings from high-throughput transcriptomic studies, clinical investigations, and mechanistic analyses, we aim to provide a coherent framework that highlights both the translational promise and the current challenges of circRNA research in T2DM. We further discuss future directions necessary to harness circRNAs for precision diagnosis, risk stratification, and therapeutic development in metabolic disease.

## 2 Biogenesis and functional characteristics of circular RNAs

Circular RNAs (circRNAs) are a distinct class of endogenous non-coding RNAs characterized by a covalently closed circular structure lacking 5′ caps and 3′ poly(A) tails. Unlike linear RNAs, circRNAs are generated through non-canonical splicing events and display unique biogenetic mechanisms and functional properties. Increasing recognition of their stability, abundance, and regulatory potential has positioned circRNAs as key components of post-transcriptional gene regulation, particularly in complex diseases such as metabolic disorders.

### 2.1 Biogenesis of circRNAs

**Figure 1:**
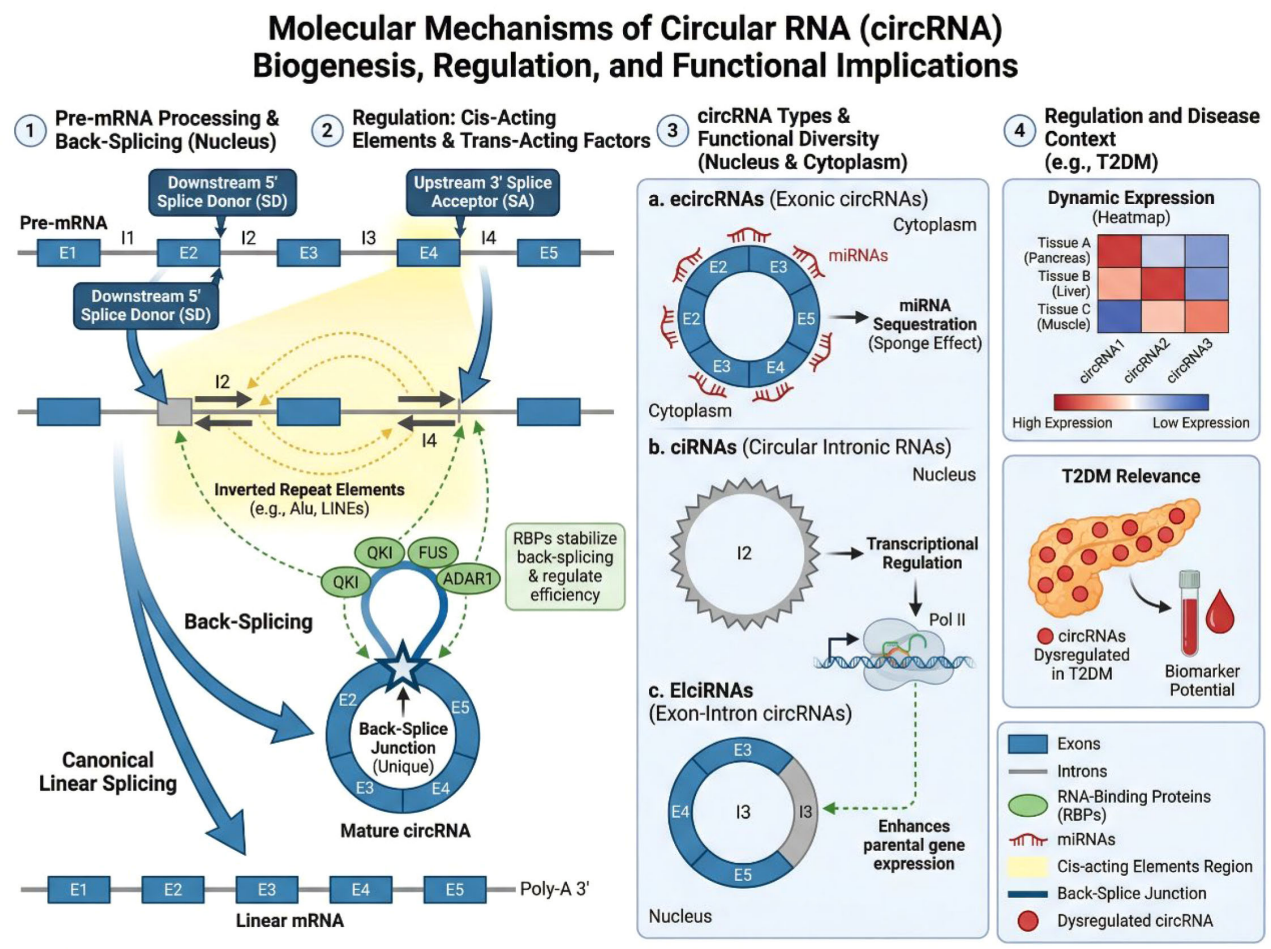
Biogenesis and types of circRNAs. The top part shows the back-splicing process of circRNA formation, highlighting the role of cis-acting elements, inverted repeats, and RNA-binding proteins. The bottom part categorizes the three main types of circRNAs: exonic circRNAs (ecircRNAs) function as miRNA sponges, intronic circRNAs (ciRNAs) regulate transcription, and exon-intron circRNAs (EIciRNAs) modulate gene expression through interactions with transcriptional machinery.

CircRNAs are primarily produced through a process known as back-splicing, in which a downstream 5′ splice donor is joined to an upstream 3′ splice acceptor. This non-linear splicing event contrasts with canonical linear splicing and is facilitated by both cis-acting elements and trans-acting splicing factors. Flanking intronic complementary sequences, such as inverted repeat elements, can bring splice sites into close proximity, thereby promoting circularization. In addition, RNA-binding proteins (RBPs) can either enhance or inhibit circRNA formation by stabilizing or blocking splice site interactions (23–25). Based on their genomic origin and composition, circRNAs are commonly classified into three major categories. Exonic circRNAs (ecircRNAs) consist solely of exonic sequences and represent the most abundant circRNA species. These circRNAs are predominantly localized in the cytoplasm and are closely associated with post-transcriptional regulatory functions, including microRNA (miRNA) sequestration. Intronic circRNAs (ciRNAs) are derived from intronic regions and are often retained within the nucleus, where they may influence transcriptional activity of their parental genes. Exon–intron circRNAs (EIciRNAs) contain both exonic and intronic sequences and have been reported to modulate gene expression through interactions with transcriptional machinery. The biogenesis of circRNAs is tightly regulated and context-dependent, resulting in cell-type-, tissue-, and disease-specific expression patterns. Importantly, circRNA abundance does not necessarily correlate with that of their linear host transcripts, suggesting independent regulatory control (26–31). This selective expression profile underlies the potential of circRNAs to serve as disease-specific biomarkers and functional regulators in pathological conditions, including type 2 diabetes mellitus (**Figure 1**).

### 2.2 Functional modes of circRNAs

CircRNAs exert diverse biological functions through multiple mechanisms, among which the miRNA sponge activity is the most extensively studied and directly relevant to metabolic disease. By harboring miRNA response elements, circRNAs can competitively bind specific miRNAs, thereby reducing their availability to target messenger RNAs (mRNAs). This competing endogenous RNA (ceRNA) mechanism enables circRNAs to indirectly modulate gene expression at the post-transcriptional level (32, 33). In metabolic contexts, such circRNA–miRNA–mRNA axes have been implicated in the regulation of insulin signaling, glucose transport, lipid metabolism, inflammatory responses, and vascular homeostasis (34). The stability and cytoplasmic localization of many exonic circRNAs further enhance their capacity to function as effective miRNA sponges. Beyond miRNA sequestration, circRNAs can also interact directly with proteins. Certain circRNAs serve as protein scaffolds or decoys, facilitating or inhibiting protein–protein interactions, altering protein localization, or modulating enzymatic activity (35). Through binding RNA-binding proteins or signaling molecules, circRNAs may participate in the fine-tuning of transcriptional and post-transcriptional regulatory networks. Although less extensively characterized in metabolic diseases, protein-binding circRNAs provide an additional layer of regulatory complexity.

Importantly, the current literature may overrepresent miRNA sponging because ceRNA models are experimentally tractable and can be readily organized into linear circRNA–miRNA–mRNA axes (22, 36, 37). However, not all circRNAs are likely to function as effective miRNA sponges *in vivo*, as this depends on circRNA abundance, subcellular localization, the number and accessibility of miRNA response elements, and the stoichiometric relationship between circRNAs and their cognate miRNAs. Increasing evidence indicates that many circRNAs exert regulatory effects through direct interaction with RNA-binding proteins (RBPs), acting as scaffolds, decoys, or modulators of protein complex assembly (38–40). A representative example is circANRIL, which binds PES1 and perturbs pre-rRNA processing, illustrating that circRNA function may be mediated through protein-centered regulatory networks rather than solely through miRNA sequestration (41). In addition, some circRNAs can serve as templates for peptide translation under specific conditions, further expanding the functional repertoire of this RNA class. These observations suggest that circRNA activities should be interpreted within a broader and more context-dependent framework in T2DM.

**Figure 2:**
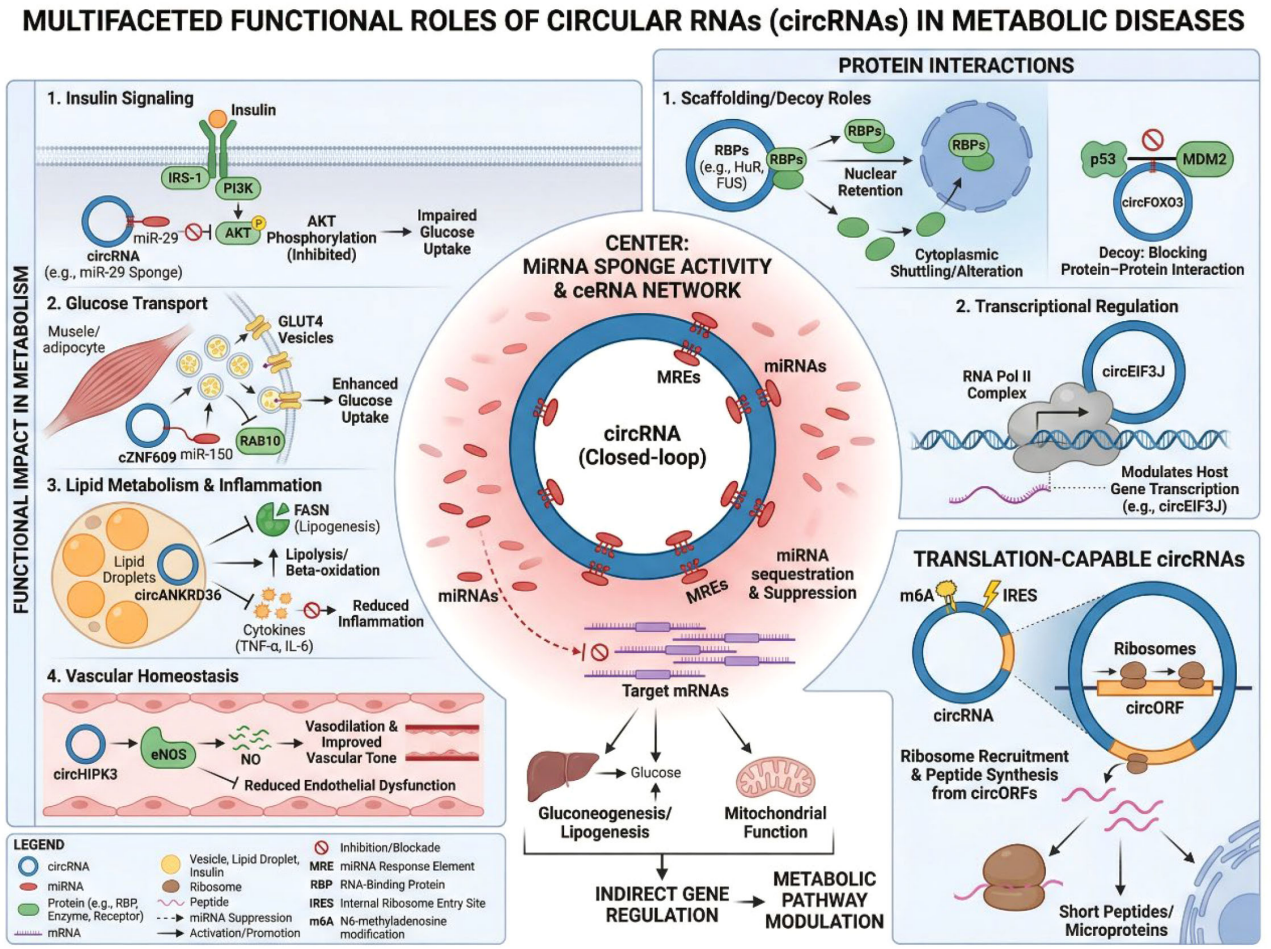
Functional modes of circRNAs. CircRNAs regulate gene expression through multiple mechanisms. In the cytoplasm, circRNAs act as miRNA sponges to modulate mRNA translation via competing endogenous RNA (ceRNA) networks, influencing metabolic and inflammatory pathways. CircRNAs can also interact with proteins as scaffolds or decoys to fine-tune regulatory signaling. In addition, a subset of circRNAs may undergo cap-independent translation to generate functional peptides in a context-dependent manner.

Emerging evidence also suggests that a subset of circRNAs possesses protein-coding potential. Despite lacking canonical translation signals, some circRNAs contain internal ribosome entry sites or N6-methyladenosine modifications that enable cap-independent translation (42–44). The functional relevance of circRNA-derived peptides remains incompletely understood and appears to be context-specific. In the setting of T2DM, translational functions of circRNAs are still largely unexplored and warrant further investigation. Collectively, the unique biogenesis and multifunctional properties of circRNAs distinguish them from other non-coding RNAs and underpin their emerging roles in disease biology. Although ceRNA-based regulation remains the most extensively characterized mechanism in T2DM, circRNAs are not functionally restricted to miRNA sequestration. Their ability to directly bind proteins, modulate protein–protein interactions, alter subcellular localization, and potentially generate bioactive peptides adds important mechanistic breadth to circRNA biology. This broader functional framework is particularly relevant to immune and inflammatory signaling, where protein-centered regulatory events may shape disease progression independently of canonical ceRNA circuits (**Figure 2**).

## 3 High-throughput profiling and ceRNA network construction in type 2 diabetes mellitus

The rapid development of high-throughput transcriptomic technologies has markedly advanced our understanding of circular RNA landscapes in type 2 diabetes mellitus (T2DM). Unlike candidate-driven approaches, unbiased profiling enables systematic identification of disease-associated circRNAs and provides a foundation for constructing regulatory networks that integrate multiple layers of RNA regulation. Such strategies have shifted circRNA research in T2DM from isolated observations toward a systems-level perspective linking transcriptomic remodeling to metabolic dysfunction.

### 3.1 High-throughput identification of circRNAs in T2DM

One of the earliest comprehensive efforts to characterize circRNA expression in T2DM employed high-throughput RNA sequencing of peripheral blood samples, revealing extensive dysregulation of non-coding RNA transcripts in diabetic patients (45–49). In this study, a large number of circRNAs were identified as differentially expressed between individuals with T2DM and healthy controls, highlighting the extensive dysregulation of circular RNAs in systemic metabolic alterations. Importantly, the authors simultaneously profiled lncRNAs, miRNAs, and mRNAs, enabling integrative analysis of multilayered RNA interactions rather than isolated transcript changes.

Functional annotation of differentially expressed circRNAs and their associated target genes through Gene Ontology (GO) and Kyoto Encyclopedia of Genes and Genomes (KEGG) enrichment analyses revealed significant clustering within pathways central to T2DM pathogenesis. Enriched biological processes included insulin signaling, glucose and lipid metabolism, inflammatory responses, and immune regulation. These findings supported the notion that circRNA dysregulation is not merely an epiphenomenon of hyperglycemia but is closely linked to core metabolic and inflammatory pathways driving disease progression (50).

Building upon these datasets, the authors constructed a comprehensive lncRNA–circRNA–miRNA–mRNA interaction network based on predicted miRNA response elements and expression correlations (51). This network-based framework illustrated how circRNAs may act as key regulatory nodes within competing endogenous RNA (ceRNA) systems, coordinating the expression of multiple genes involved in metabolic homeostasis. Notably, several circRNAs exhibited high network connectivity, suggesting potential hub roles in modulating insulin resistance and inflammatory signaling (52). Such high-throughput identification and integrative network construction provided an essential resource for subsequent functional studies and biomarker discovery in T2DM.

### 3.2 ceRNA network-based insights into metabolic regulation

While early transcriptomic studies primarily focused on identifying differentially expressed circRNAs, more recent work has emphasized the functional interpretation of circRNA-centered regulatory networks across metabolic disease spectra. In particular, systems-level analyses integrating obesity and T2DM cohorts have offered new insights into how circRNA-mediated ceRNA networks evolve along the continuum from metabolic stress to overt diabetes (53).

By analyzing transcriptomic profiles from individuals with obesity-associated metabolic dysfunction and T2DM, recent studies have demonstrated that circRNA-centered ceRNA networks are dynamically remodeled during disease progression (54). These analyses revealed that alterations in circRNA expression are closely coupled with changes in miRNA availability and downstream mRNA targets, forming coordinated regulatory modules associated with insulin resistance, lipid handling, mitochondrial function, and inflammatory signaling (55–60). Importantly, such network perturbations were not uniformly distributed but instead clustered into pathway-specific modules, reinforcing the concept that circRNAs participate in distinct metabolic programs rather than acting as global regulators. The integration of ceRNA network architecture with clinical and metabolic phenotypes further strengthened the biological relevance of these findings. Network modules enriched for circRNAs linked to insulin signaling pathways correlated with measures of glucose intolerance and insulin resistance, whereas modules associated with inflammatory and lipid metabolic pathways aligned with obesity-related traits and vascular risk. These observations underscore the capacity of ceRNA network analysis to bridge transcriptomic alterations with functional metabolic outcomes.

Collectively, high-throughput transcriptomic profiling has provided a systems-level view of circRNA-mediated regulatory networks in T2DM. By moving beyond single-molecule analyses, ceRNA-based frameworks capture the complexity of post-transcriptional regulation underlying metabolic dysfunction. Such approaches not only facilitate the identification of candidate circRNAs for diagnostic and prognostic applications but also offer mechanistic insight into how dysregulated RNA networks contribute to insulin resistance, inflammation, and metabolic disease progression. As a result, high-throughput ceRNA network analyses have become a critical foundation for understanding the emerging roles of circRNAs in T2DM and for guiding subsequent translational and functional investigations.

## 4 Circular RNAs as diagnostic biomarkers for type 2 diabetes mellitus

The exceptional stability and detectability of circular RNAs in peripheral circulation have positioned them as promising non-invasive biomarkers for type 2 diabetes mellitus (T2DM). Unlike traditional biochemical indices that primarily reflect glycemic status, circRNAs capture upstream transcriptomic and regulatory alterations associated with metabolic dysfunction. Accumulating evidence indicates that specific circRNAs are differentially expressed in blood-derived samples of individuals with T2DM and may provide diagnostic, discriminatory, and prognostic value across disease stages and complications. However, the current evidence base should be interpreted with caution. Although multiple circRNAs have shown encouraging discriminatory performance in individual studies, the overall strength of evidence remains uneven. Most published studies are based on relatively small cohorts, cross-sectional designs, and single-center populations, with limited external validation. In addition, because most available studies have been conducted in single-population cohorts, the ethnic robustness and cross-population reproducibility of reported circRNA biomarkers remain largely unresolved. In addition, substantial heterogeneity exists in sample type, patient selection, glycemic stage, analytical platform, normalization strategy, and statistical modeling, all of which may influence the reproducibility and comparability of reported diagnostic performance. Therefore, circRNAs should currently be considered emerging candidate biomarkers rather than clinically established diagnostic tools, and their translational value will depend on rigorous validation in large, independent, and prospectively characterized cohorts.

### 4.1 circRNAs for early detection and diagnosis of T2DM

Early identification of individuals at risk for T2DM remains a major unmet clinical need. Several circulating circRNAs have been reported to distinguish patients with T2DM from normoglycemic controls and, importantly, to discriminate impaired glucose regulation from overt diabetes (61). Among the earliest reported candidates, hsa_circ_0054633 was identified in peripheral blood as a putative biomarker for both pre-diabetes and T2DM (62). Expression levels of hsa_circ_0054633 were reported to be elevated in individuals with impaired fasting glucose and further increased in patients with T2DM (63), suggesting a possible association with progressive metabolic dysregulation. ROC-based analyses in these initial studies indicated potentially favorable discriminatory performance. However, such findings should be interpreted with caution and should not be taken as evidence that a single circRNA marker can outperform established clinical standards. In current practice, diagnostic frameworks centered on conventional indices such as HbA1c, fasting plasma glucose, and related metabolic measures remain the benchmark, and the incremental value of hsa_circ_0054633 beyond these standards has not yet been adequately established. Moreover, the available evidence derives primarily from relatively small Chinese cohorts and has not yet been validated across large, ethnically diverse populations. This limitation is particularly important because biomarker expression patterns in T2DM may be influenced by population-specific genetic architecture, including susceptibility loci identified by GWAS such as TCF7L2. Therefore, hsa_circ_0054633 should currently be regarded as an early candidate biomarker that may complement, rather than replace, existing diagnostic frameworks.

**Figure 3:**
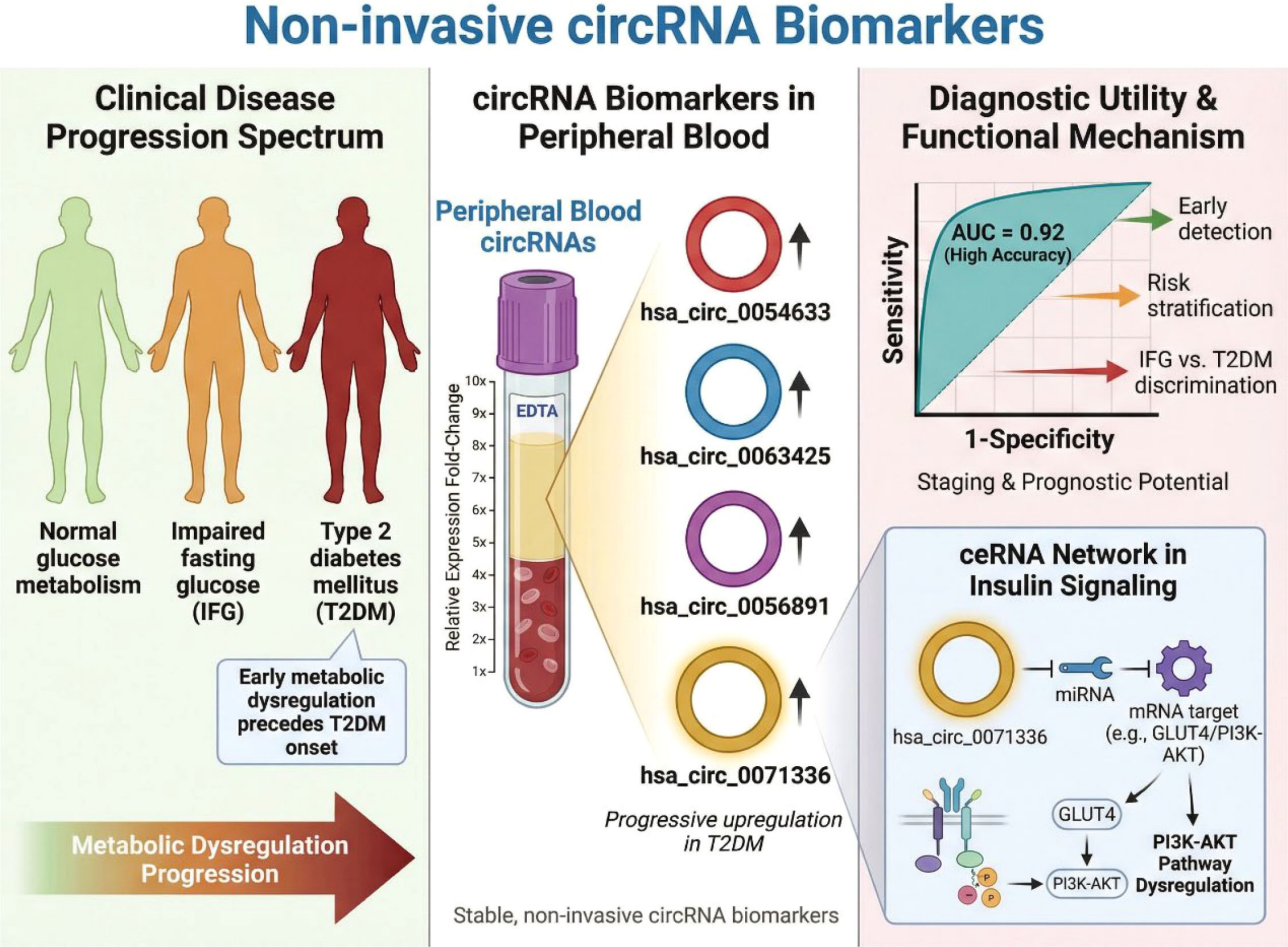
Circulating circRNAs for early detection and diagnosis of T2DM. Several circRNAs detectable in peripheral blood, including hsa_circ_0054633, hsa_circ_0063425, hsa_circ_0056891, and hsa_circ_0071336, exhibit progressive dysregulation from normoglycemia to impaired fasting glucose and overt T2DM. ROC analyses demonstrate strong diagnostic performance. These stable circulating circRNAs enable early detection, disease staging, and risk stratification, providing molecular information beyond conventional glycemic markers.

Subsequent studies expanded the repertoire of diagnostic circRNAs by identifying hsa_circ_0063425 and hsa_circ_0056891 as novel circulating biomarkers for T2DM (64). These circRNAs were detected in blood samples and exhibited significant differential expression between diabetic patients and controls. Importantly, ROC analyses revealed robust area under the curve (AUC) values, indicating strong diagnostic accuracy. Functional annotation suggested that these circRNAs were linked to insulin signaling–related pathways, including PI3K–AKT signaling, thereby providing mechanistic plausibility for their association with metabolic dysfunction (65–67). More recently, hsa_circ_0071336 has attracted attention for its ability to distinguish between impaired fasting glucose and established T2DM. Detected in peripheral blood, hsa_circ_0071336 levels were significantly altered across different glycemic states and correlated with indices of insulin resistance. Diagnostic modeling demonstrated that this circRNA could effectively differentiate early metabolic impairment from overt diabetes, underscoring its potential clinical value in disease staging. As summarized in **Figure 3**, currently reported circulating circRNA biomarkers do not represent a single uniform diagnostic signal, but rather a progressive spectrum of dysregulation across the transition from normoglycemia to impaired fasting glucose and overt T2DM. The figure highlights representative candidates, including hsa_circ_0054633, hsa_circ_0063425, hsa_circ_0056891, and hsa_circ_0071336, and emphasizes that their potential value lies in early detection, disease staging, and risk stratification rather than in standalone replacement of established glycemic testing. Notably, the inclusion of hsa_circ_0071336 also illustrates how biomarker discovery may intersect with mechanistic relevance through its reported link to glucose transporter regulation via a ceRNA axis.

Collectively, these studies suggest that circulating circRNAs may serve as candidate indicators for the early detection and diagnosis of T2DM. Nevertheless, the currently available evidence remains preliminary rather than definitive. Most diagnostic studies have evaluated circRNAs in relatively small and geographically restricted cohorts, and many have relied on retrospective or cross-sectional designs without independent external validation. In addition, the generalizability of currently reported circRNA biomarkers across ethnic groups remains uncertain. Given that T2DM susceptibility and transcriptomic regulation may be shaped by population-specific genetic backgrounds, including GWAS-associated variants such as TCF7L2, biomarker performance observed in one ethnic population should not be assumed to apply uniformly to others. In several cases, ROC-based performance appears encouraging, but the incremental value of these circRNAs over conventional clinical markers such as fasting plasma glucose, HbA1c, or HOMA-IR has not been adequately established. Moreover, differences in sample source, disease stage definition, and analytical workflows limit direct comparison across studies. These issues suggest that the current literature is more supportive of biomarker discovery than immediate clinical implementation. Future work should prioritize multicenter validation, standardized assay pipelines, and combined diagnostic models integrating circRNAs with established metabolic indicators.

### 4.2 circRNAs for disease stratification and metabolic complications

Beyond disease diagnosis, circRNAs have demonstrated potential value in stratifying T2DM patients according to complication risk and comorbid conditions. Given the heterogeneous nature of T2DM, biomarkers that reflect specific pathological trajectories, such as cardiovascular or vascular complications, are of considerable clinical importance. Hsa-circRNA11783–2 was identified as a circulating circRNA associated with both coronary artery disease and T2DM (68). Elevated expression levels of this circRNA in peripheral blood were correlated with the presence of cardiovascular comorbidity, suggesting that hsa-circRNA11783–2 may reflect shared pathogenic mechanisms linking metabolic dysfunction and vascular injury. Although initial studies highlighted its diagnostic and stratification potential, subsequent methodological discussions emphasized the importance of rigorous validation and standardized analytical pipelines, underscoring broader challenges in circRNA biomarker research.

**Figure 4:**
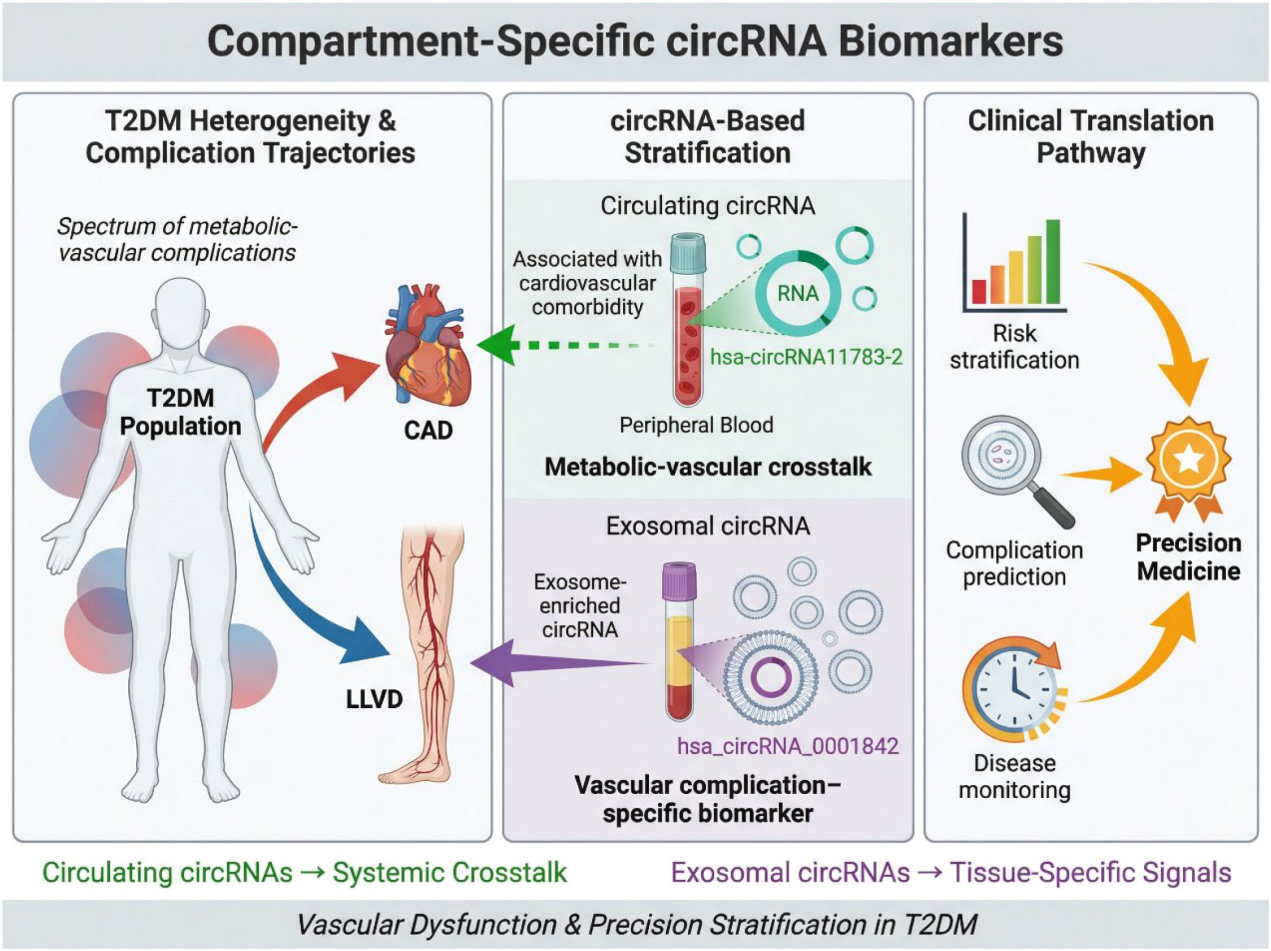
CircRNAs for disease stratification and metabolic complications in T2DM. Circulating and exosomal circRNAs enable stratification of patients with type 2 diabetes mellitus according to vascular complication risk. Peripheral blood hsa-circRNA11783–2 is associated with cardiovascular comorbidity, whereas serum exosomal hsa_circRNA_0001842 correlates with lower limb vascular disease. These compartment-specific circRNA signatures support multidimensional risk assessment and precision management beyond binary disease diagnosis.

The emergence of extracellular vesicle research has further expanded the diagnostic landscape of circRNAs. Serum exosomal hsa_circRNA_0001842 was reported as a potential biomarker for lower limb vascular disease in patients with T2DM (69). Encapsulation within exosomes confers additional stability and may enhance the biological relevance of circRNAs by reflecting active intercellular communication. Elevated levels of exosomal hsa_circRNA_0001842 were associated with vascular complications, highlighting its utility for complication-specific risk assessment rather than general disease diagnosis. The progression from whole blood to serum and, ultimately, to exosome-derived circRNAs reflects a broader evolution in biomarker discovery strategies. While early studies focused on readily accessible blood samples, more recent work suggests that compartment-specific circRNAs, particularly those enriched in extracellular vesicles, may offer greater specificity for pathological processes such as endothelial dysfunction and vascular remodeling (70–75). Importantly, the diagnostic scope of circRNAs in T2DM has expanded from binary disease classification toward multidimensional stratification. CircRNA biomarkers are increasingly being evaluated for their ability to distinguish disease stages, predict complication risk, and potentially monitor disease progression. This transition aligns with the goals of precision medicine, where molecular markers inform individualized risk assessment and clinical decision-making. As illustrated in **Figure 4**, the diagnostic scope of circRNAs in T2DM extends beyond binary disease identification toward complication-oriented stratification. The figure contrasts circulating and exosomal biomarkers in different biological compartments, highlighting peripheral blood hsa-circRNA11783–2 in association with cardiovascular comorbidity and serum exosomal hsa_circRNA_0001842 in relation to lower limb vascular disease. This comparison underscores the concept that compartment-specific circRNA signatures may provide more refined clinical information for risk assessment and precision management than a single undifferentiated biomarker framework.

Taken together, circulating and exosomal circRNAs have shown potential not only for T2DM diagnosis but also for disease stratification and complication-oriented risk assessment. However, the strength of evidence varies substantially among reported candidates. Some circRNAs appear to be associated with specific vascular or cardiovascular phenotypes, yet these observations are often derived from limited cohorts and require careful interpretation because complication status, medication exposure, renal function, and other metabolic comorbidities may act as important confounders. In addition, exosomal circRNAs may offer greater biological specificity, but their clinical application is constrained by technical variability in extracellular vesicle isolation, quantification, and normalization. Thus, while these findings are promising, they should currently be regarded as hypothesis-generating and translationally exploratory. Robust prospective studies with standardized methodologies and direct comparison against existing risk markers will be necessary before circRNA-based stratification tools can be considered clinically actionable. To provide a more critical overview of the current evidence, the key circRNAs reported for T2DM diagnosis or stratification are summarized in **Table 1**, including their sample sources, expression patterns, reported targets, quantitative diagnostic performance, and major study limitations.

**Table 1:** Circulating circular RNAs as diagnostic and stratification biomarkers in type 2 diabetes mellitus.

circRNA	Sample type	Study population	Clinical application	Diagnostic performance	Associated pathways/notes	Reference
hsa_circ_0054633	Peripheral blood	Pre-diabetes, T2DM, healthy controls	Early detection of pre-diabetes and T2DM	ROC analysis showed good diagnostic accuracy (AUC >0.80 in original study)	Gradual increase from pre-diabetes to T2DM; reflects early metabolic dysregulation	Acta Diabetol, 2017
hsa_circ_0054633	Serum	T2DM patients and controls	Diagnosis and correlation with clinical features	Positively correlated with fasting glucose and HbA1c	Confirms reproducibility across sample types	Ann Clin Lab Sci, 2021
hsa_circ_0063425	Peripheral blood	T2DM patients and controls	Diagnosis of T2DM	High diagnostic accuracy based on ROC analysis	Linked to insulin signaling pathways (e.g., PI3K–AKT)	J Clin Endocrinol Metab, 2021
hsa_circ_0056891	Peripheral blood	T2DM patients and controls	Diagnosis of T2DM	Robust AUC values in ROC analysis	Associated with metabolic regulation and insulin resistance	J Clin Endocrinol Metab, 2021
hsa_circ_0071336	Peripheral blood	IFG, T2DM, healthy individuals	Discrimination between IFG and T2DM	Strong discriminatory ability between glycemic states	Mechanistically linked to miR-93-5p/GLUT4 axis	FASEB J, 2022
hsa-circRNA11783-2	Peripheral blood	T2DM with or without coronary artery disease	Disease stratification and cardiovascular risk	Differential expression between CAD and non-CAD groups	Reflects shared metabolic–vascular pathology; methodological limitations noted	Diab Vasc Dis Res, 2017
hsa_circRNA_0001842	Serum exosomes	T2DM patients with or without lower limb vascular disease	Identification of vascular complications	Significantly elevated in patients with LLVD	Exosome-enriched circRNA; potential marker of endothelial dysfunction	J Genet, 2023
hsa_circ_0063425/hsa_circ_0056891 (panel)	Peripheral blood	T2DM cohort	Combined diagnostic panel	Improved diagnostic performance compared with single markers	Supports multi-circRNA biomarker strategy	J Clin Endocrinol Metab, 2021

## 5 ceRNA regulatory axes of circRNAs in metabolic dysfunction

Beyond their diagnostic potential, circRNAs have emerged as active regulators of metabolic homeostasis through competing endogenous RNA (ceRNA) mechanisms. By sequestering specific microRNAs, circRNAs modulate downstream gene expression programs that govern insulin signaling, β-cell function, inflammatory responses, and vascular integrity. Increasing evidence suggests that circRNA-mediated ceRNA axes are organized into discrete pathological modules that collectively drive metabolic dysfunction in type 2 diabetes mellitus (T2DM). Nevertheless, the current emphasis on ceRNA networks should not be interpreted as evidence that miRNA sponging is the exclusive or even dominant mode of circRNA action in all diabetic contexts. In practice, ceRNA mechanisms are often prioritized because they are experimentally accessible and easier to organize into linear regulatory axes. However, circRNAs may also exert non-ceRNA effects through direct interactions with RNA-binding proteins, signaling adaptors, transcriptional regulators, or enzymatic complexes. These protein-centered mechanisms are especially relevant in inflammatory and immune pathways, where circRNAs may function as scaffolds, decoys, or modulators of signaling assembly independently of miRNA sequestration. Therefore, the ceRNA framework presented in this section should be viewed as a major but incomplete representation of circRNA biology in T2DM. This also reflects a broader limitation of the field: non-ceRNA mechanisms of circRNAs in T2DM remain underexplored compared with the abundance of sponge-based studies. Therefore, the following sections emphasize ceRNA axes not because alternative mechanisms are unimportant, but because they currently constitute the best-characterized body of evidence in diabetic metabolic dysfunction.

### 5.1 Insulin resistance and glucose uptake

Insulin resistance (IR) is considered the hallmark of type 2 diabetes mellitus (T2DM) and is characterized by reduced sensitivity of peripheral tissues (such as muscle, adipose tissue, and liver) to insulin. This impaired insulin signaling leads to a range of metabolic disturbances, including defective glucose uptake, altered lipid metabolism, and inflammation (52, 76–79). The molecular mechanisms underlying IR are complex and involve multiple signaling pathways, including defects in insulin receptor substrate (IRS) signaling, PI3K-AKT activation, and GLUT4 translocation (80, 81). Among the various molecular regulators, circular RNAs (circRNAs) have emerged as key players in modulating these pathways.

A well-characterized example of circRNA involvement in IR is the hsa_circ_0071336/miR-93-5p/GLUT4 pathway (82). hsa_circ_0071336, identified as a circulating circRNA, has been shown to be differentially expressed across individuals with normoglycemia, impaired fasting glucose (IFG), and T2DM. In particular, hsa_circ_0071336 expression levels were found to increase with the progression from normal glucose tolerance to pre-diabetes and overt T2DM, suggesting that it could serve as an early indicator of metabolic disturbance. Functionally, hsa_circ_0071336 acts as a molecular sponge for miR-93-5p, a microRNA known to regulate the expression of GLUT4, the major insulin-responsive glucose transporter. MiR-93-5p has been shown to directly repress GLUT4 expression by binding to its 3′ untranslated region (UTR), thereby inhibiting glucose uptake in insulin-sensitive tissues (83). By sequestering miR-93-5p, hsa_circ_0071336 prevents the miRNA from binding to GLUT4 mRNA, thus relieving the repression and facilitating GLUT4 expression and translocation to the cell membrane in response to insulin stimulation (84). This action directly enhances glucose uptake, which is impaired in IR. The GLUT4 translocation mechanism is crucial for insulin-mediated glucose uptake in skeletal muscle and adipose tissue. In healthy individuals, insulin binding to its receptor activates the IRS-PI3K-AKT pathway, leading to the translocation of GLUT4-containing vesicles to the plasma membrane, allowing glucose to enter the cells. In IR, however, this signaling is impaired, and GLUT4 fails to efficiently localize to the membrane, resulting in reduced glucose uptake. Dysregulation of circRNA–miRNA–mRNA axes like hsa_circ_0071336/miR-93-5p/GLUT4 directly contributes to this defect in insulin-mediated glucose handling.

**Figure 5:**
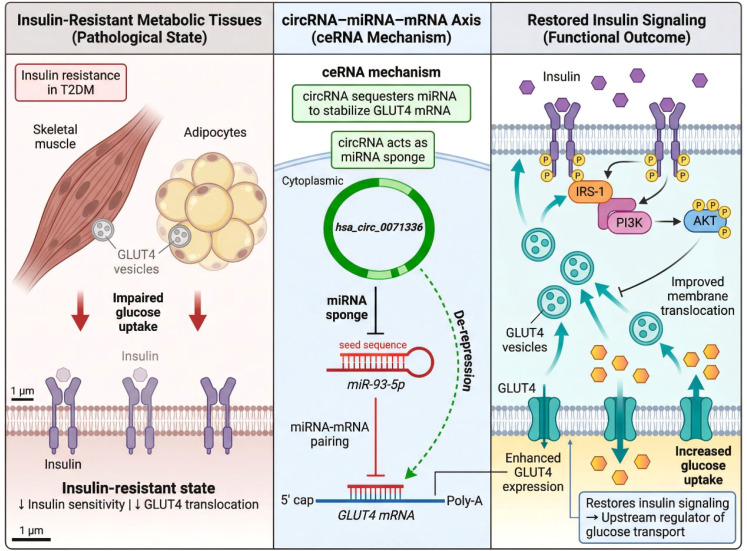
circRNA-mediated regulation of insulin resistance and glucose uptake in T2DM. Hsa_circ_0071336 acts as a competing endogenous RNA by sponging miR-93-5p, thereby relieving miRNA-mediated repression of GLUT4. This circRNA–miRNA–mRNA axis enhances GLUT4 expression and membrane translocation through insulin signaling pathways, promoting glucose uptake in insulin-sensitive tissues. Dysregulation of this mechanism contributes to impaired insulin responsiveness and metabolic dysfunction in type 2 diabetes mellitus.

Experimental studies have further validated the functional relevance of hsa_circ_0071336 in regulating GLUT4. *In vitro* cell models of insulin resistance, modulation of hsa_circ_0071336 expression led to changes in GLUT4 protein levels and its subcellular localization. Overexpression of hsa_circ_0071336 promoted GLUT4 membrane translocation, whereas knockdown of hsa_circ_0071336 resulted in a reduction in GLUT4 expression and glucose uptake, providing direct evidence for its role in regulating insulin-mediated glucose transport (85–89). These findings support the hypothesis that circRNAs, particularly hsa_circ_0071336, act as upstream regulators of glucose transport mechanisms, affecting both the availability of GLUT4 and its membrane localization in response to insulin signaling. This circRNA-mediated ceRNA axis integrates transcriptional alterations at the RNA level with functional metabolic outcomes at the cellular level. It bridges upstream transcriptomic changes with downstream defects in glucose uptake, providing a direct link between circRNA expression and insulin resistance. Unlike traditional biomarkers, which often reflect disease progression or tissue damage, circRNAs like hsa_circ_0071336 actively regulate the very processes that underlie metabolic dysfunction in T2DM. This underscores their potential as active modulators in disease progression, rather than passive bystanders or markers of disease states. Moreover, the role of circRNAs in insulin resistance highlights a broader potential for their involvement in other key metabolic processes such as lipid metabolism, oxidative stress, and mitochondrial function. By integrating data from high-throughput sequencing and functional studies, the hsa_circ_0071336/miR-93-5p/GLUT4 pathway exemplifies how circRNAs can serve as molecular switches that control crucial metabolic pathways in T2DM. **Figure 5** schematically summarizes how circRNA dysregulation may contribute to insulin resistance at the level of glucose transporter control. Specifically, hsa_circ_0071336 is presented as a representative circRNA that modulates the miR-93-5p/GLUT4 axis, thereby influencing GLUT4 expression and membrane translocation in insulin-responsive tissues. By linking ceRNA regulation to impaired glucose uptake, this figure highlights a mechanistic route through which circRNAs may connect transcriptomic dysregulation to one of the central metabolic defects of T2DM.

In conclusion, circRNAs like hsa_circ_0071336 are emerging as central regulators in the pathophysiology of insulin resistance, providing mechanistic insights into the molecular basis of T2DM. Their ability to modulate key pathways such as GLUT4 translocation places them at the forefront of new biomarker discovery and potential therapeutic strategies targeting insulin resistance.

### 5.2 Pancreatic β-cell function and survival

Pancreatic β-cell dysfunction and loss are key determinants in the pathogenesis of type 2 diabetes mellitus (T2DM). β-cell failure leads to the inability to produce adequate amounts of insulin, contributing to sustained hyperglycemia, which in turn exacerbates insulin resistance and promotes further β-cell damage. This progressive loss of β-cell function is associated with chronic metabolic stress, glucotoxicity, lipotoxicity, and oxidative stress. Therefore, maintaining β-cell function and survival is essential for preventing or delaying the onset of T2DM (90–94). Recent studies have highlighted the importance of circular RNAs (circRNAs) as regulators of β-cell metabolism and survival through competing endogenous RNA (ceRNA) mechanisms. In addition to ceRNA-based studies performed in cellular models, evidence from human primary islets has further strengthened the relevance of circRNAs to β-cell dysfunction in T2DM. A human islet-focused analysis identified more than 2, 600 circRNAs in pancreatic islets and showed that several highly abundant circRNAs, including circCAMSAP1, were associated with T2D status. Notably, circCAMSAP1 was not only abundant in human islets but was also detectable in peripheral blood, supporting the concept that islet-enriched circRNAs may have translational value as circulating biomarkers (95). Although the strongest functional association with insulin secretory index in that study was reported for circCIRBP rather than circCAMSAP1, the identification of circCAMSAP1 across both islet and blood compartments remains important because it links tissue-level circRNA dysregulation with clinical biomarker accessibility. These findings broaden the discussion of β-cell-related circRNAs beyond single ceRNA axes and underscore the importance of incorporating human islet-derived evidence into mechanistic and translational reviews of T2DM.

A key example of circRNA involvement in β-cell function is the hsa_circ_0115355/miR-145/SIRT1 axis. hsa_circ_0115355 was first identified as a circRNA dysregulated in patients with T2DM and is functionally linked to β-cell performance (96). CircRNAs, including hsa_circ_0115355, exert their effects by sponging specific microRNAs (miRNAs), thereby modulating the availability of miRNAs to regulate target mRNAs. In this case, hsa_circ_0115355 acts as a molecular sponge for miR-145, a miRNA previously implicated in the regulation of insulin production and β-cell survival. By sequestering miR-145, hsa_circ_0115355 prevents miR-145 from repressing its downstream target, Sirtuin 1 (SIRT1), a critical regulator of cellular metabolism, mitochondrial function, and stress resistance. SIRT1 plays a crucial role in promoting β-cell survival and function by enhancing insulin secretion, protecting against glucotoxicity, and mitigating oxidative stress (97–100). SIRT1 modulates a wide array of cellular processes, including mitochondrial biogenesis, protein deacetylation, and the activation of stress-responsive pathways. In the context of β-cells, SIRT1 facilitates adaptive responses to metabolic stress, supporting β-cell longevity under conditions of high glucose and free fatty acid exposure (99, 101–103). In addition to its role in β-cell protection, SIRT1 has been shown to enhance insulin secretion by increasing insulin gene transcription and promoting the expression of insulin-processing enzymes. Through the hsa_circ_0115355/miR-145/SIRT1 ceRNA axis, hsa_circ_0115355 contributes to β-cell metabolic resilience. By modulating the miRNA-mediated repression of SIRT1, hsa_circ_0115355 helps sustain β-cell function under stressful conditions. This circRNA-mediated regulation enables β-cells to better withstand glucotoxicity, lipotoxicity, and oxidative stress, which are hallmark stressors in T2DM pathogenesis. Loss of this regulatory circuit due to circRNA dysregulation has been associated with impaired β-cell function, as reduced SIRT1 activity leads to β-cell apoptosis, insulin secretion defects, and eventual β-cell failure.

**Figure 6:**
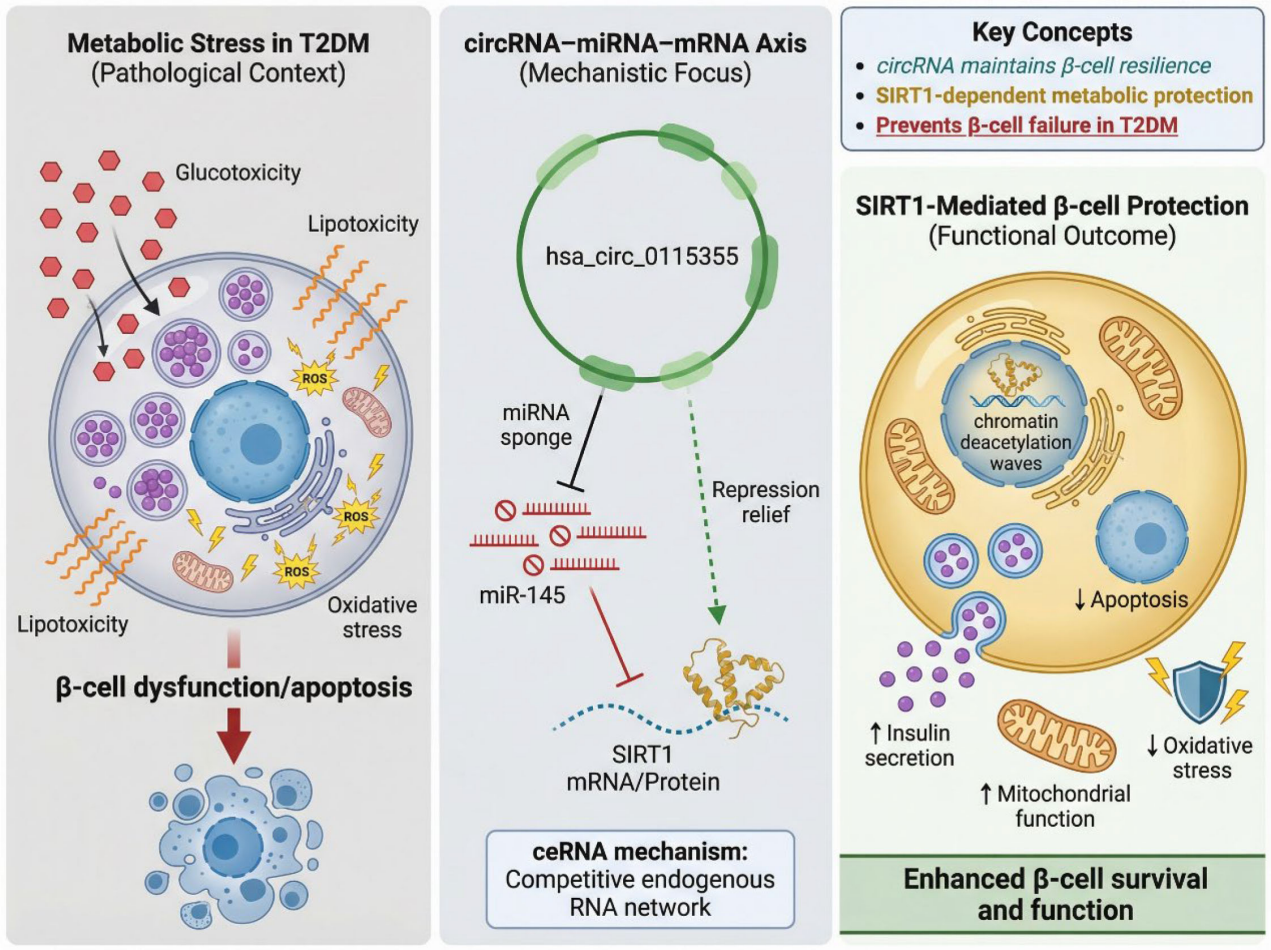
circRNA-mediated regulation of pancreatic β-cell function and survival in T2DM. Hsa_circ_0115355 functions as a competing endogenous RNA by sponging miR-145, thereby relieving miRNA-mediated repression of SIRT1. Activation of SIRT1 enhances insulin secretion, improves mitochondrial function, and protects β-cells from glucotoxicity, lipotoxicity, and oxidative stress. Dysregulation of this circRNA–miRNA–mRNA axis contributes to β-cell dysfunction and progressive β-cell loss in type 2 diabetes mellitus.

Experimental studies have provided functional validation for this circRNA–miRNA–mRNA axis. Overexpression of hsa_circ_0115355 in β-cell lines has been shown to enhance SIRT1 expression and protect cells from glucose-induced apoptosis, while knockdown of hsa_circ_0115355 resulted in reduced SIRT1 expression and increased β-cell death. These findings suggest that hsa_circ_0115355 plays a protective role in β-cell survival by maintaining optimal levels of SIRT1, thus preventing early β-cell loss in T2DM. Moreover, the therapeutic potential of modulating this axis has been explored *in vitro*, where restoring hsa_circ_0115355 expression in β-cells has been shown to ameliorate β-cell dysfunction and enhance insulin secretion. This proof-of-concept suggests that circRNAs may offer novel therapeutic avenues for protecting β-cells in T2DM (104–107). Importantly, the hsa_circ_0115355–miR-145–SIRT1 pathway is mechanistically well-defined, and its dysregulation has been implicated in the pathophysiology of T2DM. The therapeutic potential of targeting this circRNA-mediated axis extends beyond mere biomarkers, offering a potential strategy for restoring β-cell function and insulin secretion. By upregulating SIRT1 through circRNA manipulation, it may be possible to protect β-cells from metabolic stress and delay the onset of T2DM in at-risk populations. However, while these findings are promising, the therapeutic application of circRNAs as modulators of β-cell function remains in its infancy. Several challenges remain, including the need for more extensive *in vivo* validation and the development of targeted delivery methods to modulate circRNA expression specifically within β-cells. Moreover, the potential off-target effects and long-term stability of circRNA-based interventions need to be carefully evaluated before clinical translation (**Figure 6**).

In conclusion, the hsa_circ_0115355/miR-145/SIRT1 ceRNA axis represents a critical regulatory circuit that links circRNA expression to β-cell function and survival. This pathway exemplifies the emerging role of circRNAs in maintaining metabolic homeostasis and protecting insulin-producing cells under metabolic stress. Future research aimed at enhancing our understanding of circRNA-mediated regulation in β-cells will be essential for developing novel therapeutic strategies to preserve β-cell function and improve clinical outcomes in T2DM.

### 5.3 Chronic inflammation and immune–metabolic crosstalk

Chronic low-grade inflammation is now widely recognized as a central feature of type 2 diabetes mellitus (T2DM), contributing to the development of insulin resistance and exacerbating metabolic deterioration (108–112). This inflammatory response is not limited to the classical immune organs such as the spleen or lymph nodes; instead, it involves systemic interactions between immune cells, metabolic tissues, and various circulating factors. Immune cells, particularly those in peripheral blood, play an integral role in driving inflammatory stress and metabolic dysfunction through the release of pro-inflammatory cytokines and mediators (113–116). A growing body of evidence suggests that circRNAs expressed in immune cells are important regulators of the immune–metabolic interactions that sustain chronic low-grade inflammation in type 2 diabetes mellitus (51, 82, 117). In the diabetic milieu, persistent hyperglycemia, excess lipids, oxidative stress, and mitochondrial dysfunction reshape immune-cell phenotypes and promote maladaptive inflammatory signaling. CircRNAs are increasingly recognized as upstream modulators of these processes, not only through ceRNA-dependent regulation but also potentially through direct interaction with RNA-binding proteins, inflammasome-associated components, and signaling complexes. Rather than acting only as passive biomarkers, circRNAs may participate in the reprogramming of innate and adaptive immune responses that link metabolic stress to insulin resistance and tissue injury. This broader mechanistic view is particularly important in inflammatory settings, where scaffold- or decoy-like circRNA functions may shape signaling output independently of miRNA sequestration. Evidence from diabetes and other inflammatory disorders further supports the concept that circRNAs can influence macrophage activation states, inflammasome signaling, and T-cell dysfunction, although direct mechanistic validation in T2DM remains limited (118).

One representative example in T2DM is circANKRD36, which was reported to be significantly upregulated in peripheral blood leukocytes from patients with T2DM and positively correlated with inflammatory markers such as TNF-α, IL-6, and C-reactive protein (119, 120). This expression pattern suggests that circANKRD36 is associated with systemic immune activation rather than merely reflecting metabolic imbalance. Although its downstream targets in T2DM have not yet been fully defined, available data indicate that circANKRD36 may sponge inflammation-related miRNAs and thereby enhance pro-inflammatory gene expression programs. This observation is particularly relevant because monocytes and macrophages are central mediators of metabolic inflammation in obesity-associated insulin resistance and T2DM (121, 122). A sustained shift toward an M1-like macrophage phenotype amplifies the production of IL-1β, IL-6, and TNF-α, whereas impaired M2 polarization weakens inflammation resolution, tissue repair, and insulin-sensitizing signaling (123, 124). In this context, circRNAs may function as rheostats of macrophage polarization by modulating miRNA-controlled pathways involved in NF-κB activity, oxidative stress responses, mitochondrial homeostasis, and cytokine production. Therefore, circANKRD36 can be discussed not only as an inflammation-associated circRNA, but also as a plausible regulator of the M1/M2 balance in diabetic immune microenvironments.

Another important layer of immune–inflammatory crosstalk involves activation of the NLRP3 inflammasome, which has a well-established role in T2DM and diabetic complications (125–127). NLRP3 activation in monocytes/macrophages promotes caspase-1 cleavage and the maturation of IL-1β and IL-18, thereby aggravating insulin resistance, β-cell stress, endothelial dysfunction, and tissue inflammation (118). Although direct evidence linking circANKRD36 to NLRP3 in T2DM is still lacking, circRNA biology provides a strong mechanistic framework for such regulation. In diabetic kidney disease, for example, circ_0004951 was shown to promote high-glucose-induced pyroptosis through the miR-93-5p/NLRP3 axis, demonstrating that circRNAs can directly modulate inflammasome activation in diabetic settings (128). Similarly, circRNA DICAR was reported to suppress diabetic pyroptosis in cardiomyocytes, supporting the broader concept that circRNAs can either amplify or restrain inflammasome-associated injury depending on context (129). These findings suggest that the inflammatory relevance of circRNAs in T2DM extends beyond cytokine correlation and may include direct control of pyroptotic and inflammasome-dependent pathways.

**Figure 7:**
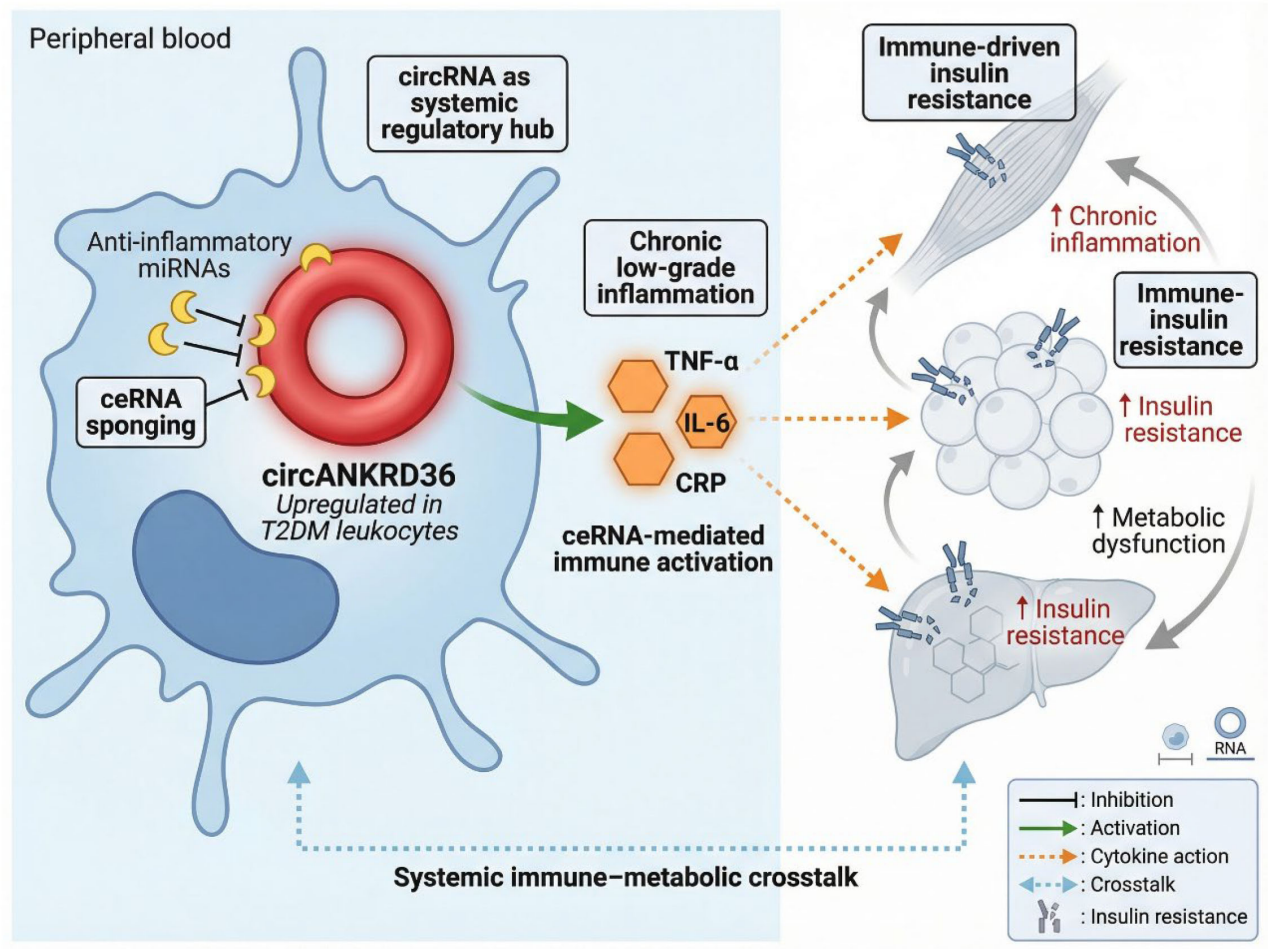
circRNA-mediated immune–metabolic crosstalk in T2DM. CircANKRD36 is upregulated in circulating immune cells of patients with type 2 diabetes mellitus and promotes chronic low-grade inflammation through ceRNA-mediated regulation of inflammatory signaling. Enhanced release of pro-inflammatory cytokines, including TNF-α and IL-6, amplifies systemic immune–metabolic crosstalk, contributing to insulin resistance and metabolic dysfunction across peripheral tissues.

In addition to innate immune signaling, adaptive immune dysfunction should also be considered in circRNA-mediated immune crosstalk. T2DM is increasingly associated with expansion of senescent and dysfunctional T-cell populations, including CD8+CD57+ senescent T cells and PD-1-expressing exhausted-like T cells, both of which contribute to chronic inflammation and impaired immune competence (130–132). Senescent T cells can produce pro-inflammatory mediators and thereby reinforce systemic insulin resistance, while exhausted T cells exhibit defective effector responses and altered immunometabolic fitness (133–135). Although circRNA-specific data in diabetic T-cell exhaustion remain scarce, evidence from broader immune biology indicates that non-coding RNAs can regulate inhibitory receptor expression, cytokine signaling, and metabolic adaptation in T cells. Accordingly, it is plausible that dysregulated circRNAs in T2DM participate in T-cell senescence or exhaustion-like states by modulating ceRNA networks linked to PD-1 signaling, mitochondrial metabolism, redox stress, and chronic antigenic or inflammatory stimulation. This possibility deserves greater attention because it would connect circRNA dysregulation not only to inflammation itself, but also to the persistence and poor resolution of immune dysfunction in diabetes (**Figure 7**).

Taken together, current evidence supports a broader model in which circRNAs contribute to immune–inflammatory crosstalk in T2DM at multiple levels: by shaping macrophage polarization, by influencing inflammasome activation and pyroptotic signaling, and potentially by sustaining T-cell senescence or exhaustion-like dysfunction. However, the strength of evidence is not yet uniform across these domains. Macrophage- and inflammasome-related mechanisms are increasingly supported by studies in diabetic tissues and complication models, whereas the involvement of circRNAs in T-cell dysfunction in T2DM remains more inferential and requires direct validation in patient-derived immune subsets. Future studies integrating single-cell transcriptomics, immune phenotyping, and circRNA perturbation experiments in monocytes/macrophages and T-cell subpopulations will be essential to define whether circRNAs such as circANKRD36 are true drivers of immune dysregulation or primarily biomarkers of inflammatory burden.

### 5.4 Vascular dysfunction and metabolic complications

Vascular complications are a leading cause of morbidity and mortality in type 2 diabetes mellitus (T2DM), contributing significantly to the development of cardiovascular disease, diabetic nephropathy, and diabetic retinopathy (136–138). These complications arise from a complex interplay of endothelial dysfunction, oxidative stress, chronic inflammation, and metabolic abnormalities. While insulin resistance and glucose toxicity are primary drivers of these processes, emerging evidence suggests that circRNAs, through their involvement in competing endogenous RNA (ceRNA) networks, also play a critical role in vascular injury and metabolic comorbidities associated with T2DM.

One well-characterized circRNA involved in vascular dysfunction is circHMGCS1. CircHMGCS1 has been shown to aggravate endothelial dysfunction under diabetic conditions through its interaction with miR-4521 (139). HMGCS1 (3-hydroxy-3-methylglutaryl-CoA synthase 1) is a key enzyme involved in cholesterol synthesis, and its dysregulation in endothelial cells is linked to lipid metabolism imbalances, which contribute to atherosclerosis and other vascular pathologies. CircHMGCS1 acts as a miRNA sponge for miR-4521, relieving the inhibition of downstream targets involved in endothelial homeostasis and oxidative stress responses. These targets include critical antioxidant enzymes and pro-inflammatory factors that regulate vascular inflammation and endothelial integrity. Experimental studies have provided functional validation for the circHMGCS1/miR-4521 axis. *In vitro*, endothelial cells exposed to high glucose conditions exhibited increased expression of circHMGCS1, along with decreased levels of miR-4521. This dysregulation resulted in enhanced oxidative stress and inflammation, exacerbating endothelial injury. Knockdown of circHMGCS1 alleviated these effects, suggesting that circHMGCS1 plays a significant role in modulating the endothelial cell response to metabolic stress in T2DM. *In vivo* studies have further confirmed that circHMGCS1-mediated regulation of oxidative stress and inflammatory pathways contributes to vascular pathology, making it a promising target for therapeutic intervention in T2DM-related vascular complications. In addition to its role in vascular dysfunction, circRNAs are increasingly recognized as important regulators of metabolic comorbidities in T2DM. One such example is the circ_0004535/miR-1827/CASP8 network, which has been implicated in T2DM-associated non-alcoholic fatty liver disease (NAFLD) (37). NAFLD is a common metabolic comorbidity of T2DM, characterized by the accumulation of fat in the liver, hepatocellular injury, and inflammation. In this context, circ_0004535 acts as a ceRNA for miR-1827, leading to the upregulation of caspase-8 (CASP8), a key mediator of apoptosis and inflammatory signaling in hepatocytes. Functional assays demonstrated that overexpression of circ_0004535 in hepatocytes resulted in enhanced CASP8 expression, promoting hepatocellular apoptosis and inflammatory cytokine release. This axis appears to be particularly relevant in the setting of T2DM, where elevated blood glucose and free fatty acids exacerbate liver injury. In contrast, inhibition of circ_0004535 reduced CASP8 expression and attenuated liver cell death, offering a potential therapeutic strategy for preventing or reversing NAFLD in diabetic patients. This mechanism also underscores the broader implications of circRNAs beyond glucose metabolism, as they contribute to the regulation of lipid metabolism and organ-specific complications in T2DM.

The circ_0004535/miR-1827/CASP8 network illustrates how circRNA-mediated ceRNA regulation extends its impact beyond glucose handling to encompass lipid dysregulation and organ-specific complications. In the case of NAFLD, circ_0004535 participates in a complex feedback loop, linking abnormal lipid accumulation to hepatic inflammation and cell death, which may further accelerate the progression of T2DM. Such findings suggest that circRNAs could serve as biomarkers or therapeutic targets for multiple organ systems, providing a comprehensive approach to managing the widespread effects of T2DM. The systemic role of circRNAs in regulating metabolic and vascular dysfunction highlights their potential as novel therapeutic targets for T2DM-related complications. By modulating circRNA expression or disrupting specific ceRNA networks, it may be possible to alleviate endothelial dysfunction, reduce oxidative stress, and prevent organ damage. Moreover, circRNAs may also serve as valuable biomarkers for predicting and monitoring the progression of T2DM-related vascular and hepatic complications. However, significant challenges remain in translating these findings into clinical practice. Much of the current research is based on cell line models or animal studies, and further *in vivo* validation in human tissues is required to confirm the therapeutic potential of targeting circRNA-mediated ceRNA networks. Additionally, the development of effective delivery methods to specifically target circRNAs in relevant tissues (such as endothelial cells or hepatocytes) remains a critical hurdle.

**Figure 8:**
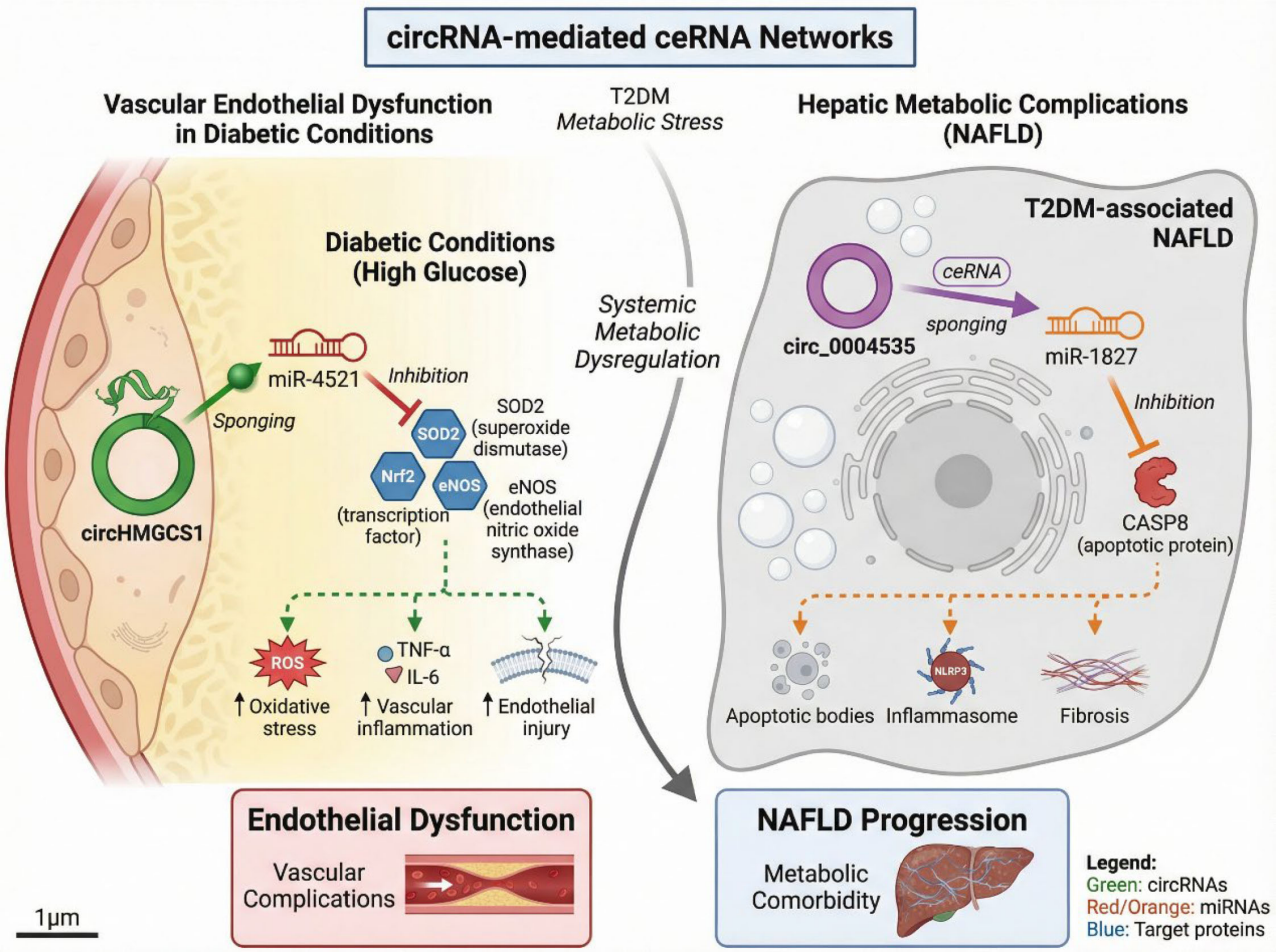
circRNA-mediated vascular dysfunction and metabolic complications in T2DM. CircRNAs contribute to organ-specific complications in type 2 diabetes mellitus through ceRNA regulation. In endothelial cells, circHMGCS1 sponges miR-4521, exacerbating oxidative stress and vascular inflammation, leading to endothelial dysfunction. In hepatocytes, circ_0004535 sequesters miR-1827 to upregulate CASP8, promoting apoptosis and inflammatory signaling and accelerating T2DM-associated non-alcoholic fatty liver disease progression.

In conclusion, circRNAs, such as circHMGCS1 and circ_0004535, play integral roles in the pathogenesis of vascular dysfunction and metabolic comorbidities in T2DM. Their regulation of oxidative stress, inflammation, and apoptosis in endothelial cells and hepatocytes underscores the broad impact of circRNA-mediated ceRNA networks in metabolic disease. Future research into circRNA-based interventions offers exciting therapeutic possibilities for the prevention and treatment of T2DM-related vascular complications and organ-specific damage (**Figure 8**) (140).

Together, these findings indicate that circRNA-driven ceRNA networks are integral to the development of vascular dysfunction and metabolic complications. Rather than acting in isolation, circRNAs orchestrate interconnected regulatory circuits that link metabolic stress to endothelial injury and multi-organ pathology.

## 6 Exosomal circRNAs and therapeutic implications

The identification of circular RNAs in extracellular vesicles has expanded their translational relevance beyond intracellular regulation. Exosomes, as nanoscale vesicles actively released by cells, protect RNA cargo from degradation and facilitate intercellular communication. The enrichment of circRNAs within exosomes provides new opportunities for biomarker development and offers preliminary insights into their potential roles as mediators or indicators of therapeutic responses in type 2 diabetes mellitus (T2DM).

### 6.1 Exosomal circRNAs as biomarkers

**Figure 9:**
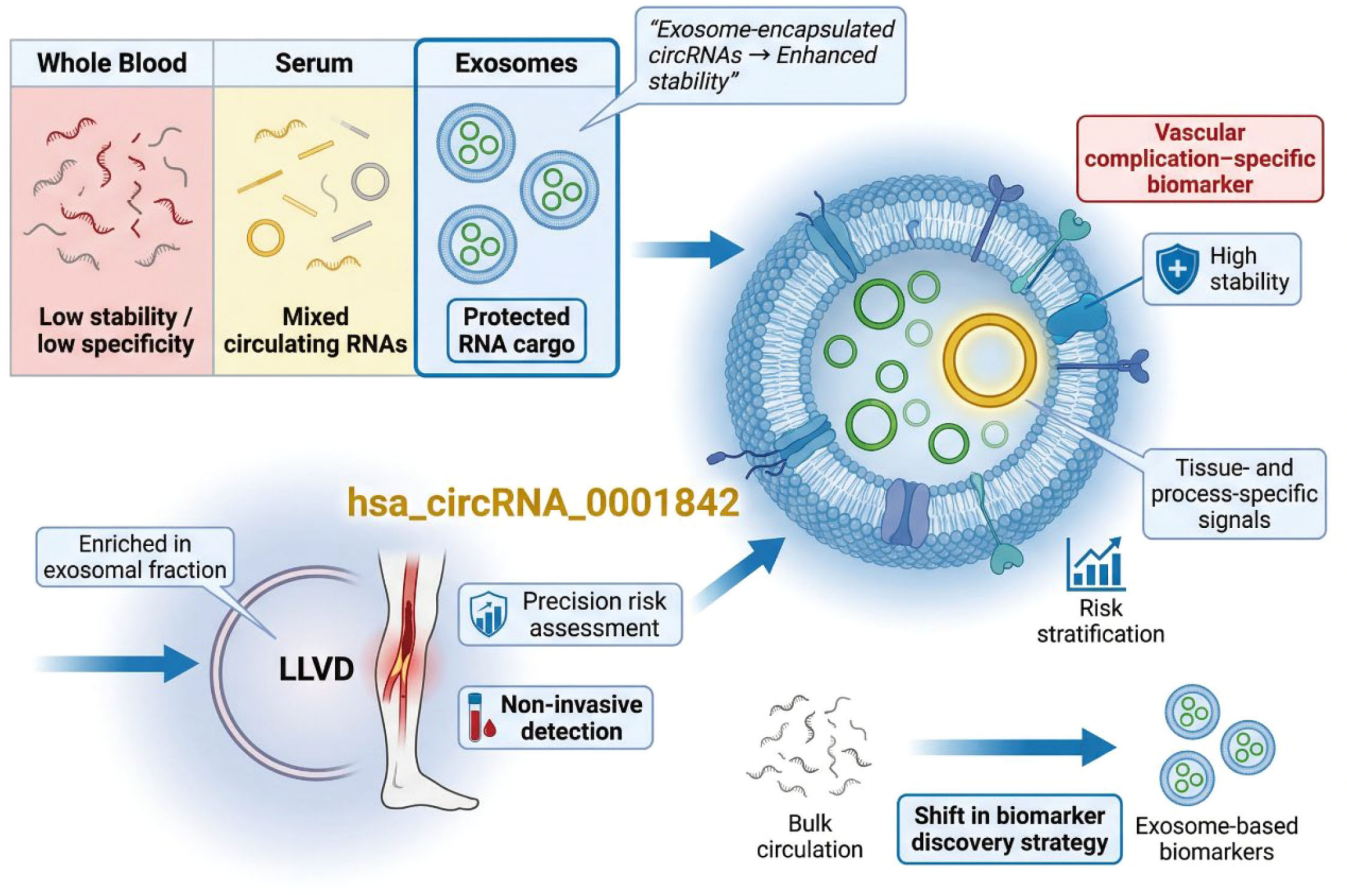
Exosomal circRNAs as compartment-specific biomarkers in T2DM. Exosome-encapsulated circRNAs exhibit enhanced stability and reflect tissue-specific pathological processes. Serum exosomal hsa_circRNA_0001842 is selectively enriched in patients with type 2 diabetes mellitus complicated by lower limb vascular disease and is associated with vascular pathology. These findings highlight exosomal circRNAs as non-invasive biomarkers for complication-specific risk stratification and precision assessment beyond bulk circulating RNAs.

Compared with freely circulating RNAs, exosome-encapsulated circRNAs exhibit enhanced stability and may better reflect pathophysiological processes occurring in specific tissues. Recent evidence supports the utility of exosomal circRNAs as biomarkers for complication-specific risk stratification in T2DM. Serum exosomal hsa_circRNA_0001842 was reported to be significantly elevated in patients with T2DM complicated by lower limb vascular disease. Importantly, this circRNA was selectively enriched in exosomal fractions rather than whole serum, highlighting the advantage of vesicle-based RNA analysis. Elevated levels of exosomal hsa_circRNA_0001842 were associated with vascular pathology, suggesting a potential role in identifying patients at increased risk of peripheral vascular complications. The detection of circRNAs within exosomes underscores a shift in biomarker discovery strategies from bulk circulation toward compartment-specific molecular signatures. Exosomal circRNAs may capture active cellular responses related to endothelial dysfunction, inflammation, or metabolic stress, thereby offering greater specificity than total circulating RNA (141). However, current evidence remains largely associative, and standardized protocols for exosome isolation, circRNA quantification, and normalization are required before clinical implementation (**Figure 9**).

### 6.2 circRNAs as therapeutic targets or response indicators

**Figure 10:**
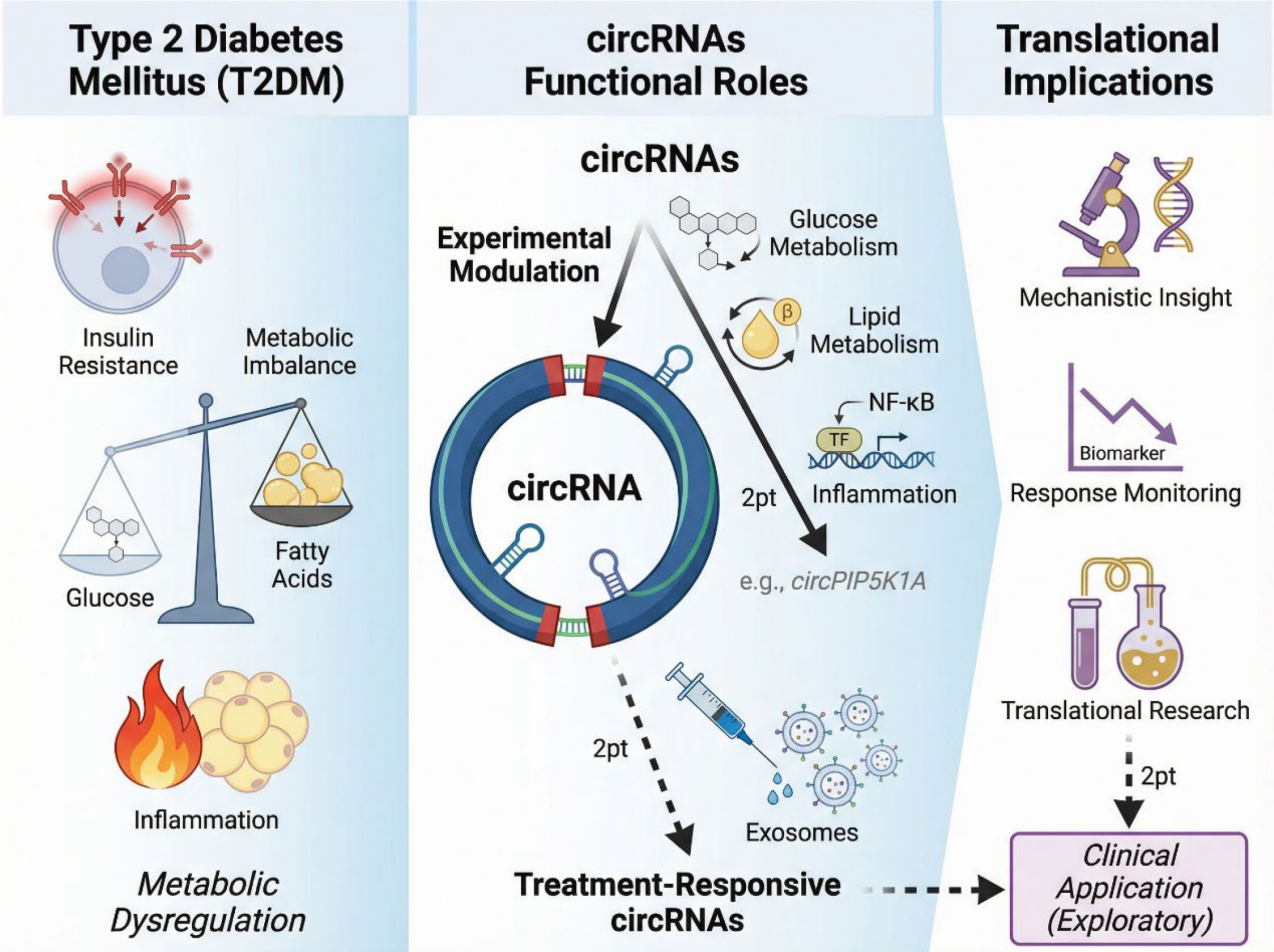
circRNAs as therapeutic targets or response indicators in T2DM. CircRNAs may function as mechanistic modulators or indicators of treatment response in type 2 diabetes mellitus. CircPIP5K1A influences glucose and lipid metabolism and inflammatory pathways in experimental models, providing proof-of-concept for circRNA modulation. In parallel, therapeutic interventions can alter exosome-derived circRNA profiles, suggesting a role for circRNAs as exploratory biomarkers for monitoring biological responses rather than established therapeutic targets.

Beyond their diagnostic value, circRNAs have been proposed as potential therapeutic targets or indicators of treatment response in metabolic disease. However, evidence supporting direct therapeutic targeting of circRNAs in T2DM remains preliminary and should be interpreted with caution. One example is circPIP5K1A, which was shown to promote glucose and lipid metabolism disorders as well as inflammatory responses in experimental models of T2DM (142). Functional manipulation of circPIP5K1A expression influenced metabolic and inflammatory phenotypes, suggesting that circRNAs may actively participate in disease-driving pathways rather than serving solely as biomarkers. These findings provide proof-of-concept that circRNA modulation can impact metabolic homeostasis, although translation into clinical therapy would require substantial advances in delivery strategies, specificity, and safety. CircRNAs have also been explored as potential indicators of therapeutic response rather than direct targets. In this context, interventions that modulate circRNA expression profiles may reflect underlying biological effects of treatment. For example, electroacupuncture was reported to alleviate metabolic dysfunction in T2DM models by promoting the expression of specific exosome-derived circRNAs (143). While such findings suggest that circRNA expression patterns may respond to therapeutic interventions, they should be viewed as exploratory. The causal contribution of circRNAs to treatment efficacy, as well as their consistency across patient populations, remains to be established. Importantly, current studies examining circRNAs in therapeutic contexts are limited by small sample sizes, heterogeneous methodologies, and a lack of long-term outcome data. Most evidence supports a role for circRNAs as biological indicators or mechanistic intermediates, rather than validated therapeutic targets (**Figure 10**). As such, circRNAs are more appropriately positioned as components of translational research pipelines that inform disease mechanisms and treatment responses, rather than as immediate candidates for clinical intervention. Overall, research on exosomal circRNAs and therapeutic implications represents an emerging frontier in T2DM biology. While early findings highlight their promise as biomarkers and mechanistic modulators, rigorous validation and cautious interpretation are essential. Future studies integrating longitudinal sampling, standardized analytical frameworks, and functional validation will be critical to determine whether circRNAs can be reliably harnessed for therapeutic monitoring or intervention in metabolic disease.

In addition to mechanistic insights, emerging studies highlight the translational potential of targeting circRNAs in diabetes and its cardiovascular complications. RNA-directed strategies such as antisense oligonucleotides (ASOs) and siRNAs are conceptually attractive because they can be designed to selectively suppress pathogenic RNA species, and broader cardiovascular research has already demonstrated the growing feasibility of RNA-based therapeutics (144). However, circRNA-directed interventions in T2DM remain largely preclinical at present, and challenges related to delivery efficiency, tissue specificity, durability, and off-target effects still limit near-term clinical translation. Exosomal circRNAs add another important layer of translational relevance. For example, serum exosomal hsa_circRNA_0001842 has been reported as a potential biomarker for lower limb vascular disease in T2DM, with an AUC of 0.79, sensitivity of 88.75%, and specificity of 68.75% in the validation cohort (69). These findings support a more integrated perspective in which circRNA-targeted strategies and extracellular circRNA biology are considered together in the translational landscape of diabetic cardiovascular complications.

## 7 Challenges and future perspectives

Despite growing interest in circular RNAs as diagnostic biomarkers and regulatory molecules in type 2 diabetes mellitus (T2DM), several conceptual and technical challenges currently limit their translational application. Addressing these issues is essential for advancing circRNA research from exploratory studies toward clinically meaningful implementation.

### 7.1 Limited sample sizes and cohort heterogeneity

A major limitation of existing circRNA studies in T2DM is the relatively small sample size of most cohorts. Many investigations rely on single-center designs with limited statistical power, increasing the risk of false-positive findings and reducing generalizability (27). Moreover, patient populations often exhibit substantial heterogeneity with respect to age, sex, ethnicity, disease duration, medication use, obesity status, and comorbid conditions. These factors can profoundly influence circRNA expression profiles but are not consistently controlled or stratified across studies. The absence of large, well-characterized cohorts complicates the interpretation of diagnostic performance metrics such as receiver operating characteristic curves and area under the curve values. Without validation in independent and diverse populations, the robustness of reported circRNA biomarkers remains uncertain. Future studies should prioritize adequately powered cohorts and harmonized clinical phenotyping to improve reproducibility and translational relevance.

### 7.2 Inconsistent circRNA annotation and nomenclature

Another critical challenge lies in the lack of standardized circRNA annotation and nomenclature. CircRNAs are frequently named according to different databases, genomic coordinates, or host genes, leading to confusion and difficulty in cross-study comparisons. In some cases, circRNAs reported under different identifiers may represent the same circular transcript, whereas similarly named circRNAs may correspond to distinct back-splicing events. This inconsistency hampers data integration, meta-analysis, and the development of unified circRNA reference resources for metabolic disease. Standardized annotation frameworks and consensus nomenclature are urgently needed to facilitate communication across studies and accelerate progress in the field.

### 7.3 Technical and statistical biases in circRNA profiling

CircRNA detection and quantification are highly dependent on analytical platforms and bioinformatic pipelines. Differences between microarray-based approaches and RNA sequencing can lead to substantial variability in circRNA identification and expression estimates. Microarrays are limited to predefined circRNA probes, whereas RNA sequencing enables discovery of novel circRNAs but is sensitive to sequencing depth, read length, and alignment algorithms. In addition, batch effects, normalization strategies, and statistical thresholds vary widely across studies, contributing to inconsistent results. Many investigations rely on differential expression analysis without rigorous correction for multiple testing or confounding variables. These technical and statistical biases underscore the need for standardized experimental and analytical workflows, as well as transparent reporting of methodological details.

### 7.4 Insufficient mechanistic validation of ceRNA interactions

Although ceRNA network analyses have provided valuable hypotheses regarding circRNA-mediated regulation, functional validation of these interactions remains limited. Many proposed circRNA–miRNA–mRNA axes are based on bioinformatic predictions and correlation analyses rather than direct experimental evidence (145). The stoichiometric requirements for effective miRNA sponging, cellular localization of circRNAs, and context-specific expression levels are often not fully addressed.

Furthermore, most mechanistic studies rely on *in vitro* models, with relatively few investigations extending findings to *in vivo* systems or human tissues. Without comprehensive validation, the causal contribution of circRNA-mediated ceRNA regulation to metabolic dysfunction remains difficult to establish. Future research should emphasize rigorous experimental validation, including loss- and gain-of-function studies, rescue experiments, and assessment of downstream metabolic phenotypes.

### 7.5 Future directions and translational perspectives

Looking forward, several strategic directions may enhance the impact of circRNA research in T2DM (146). Large-scale, multicenter, and prospective cohort studies are needed to validate candidate circRNA biomarkers across diverse populations and disease stages. Longitudinal sampling would further enable assessment of circRNA dynamics during disease progression and treatment. Integration of circRNA profiling with emerging high-resolution technologies, such as single-cell and spatial transcriptomics, offers exciting opportunities to delineate cell-type-specific and tissue-specific circRNA functions. Such approaches may clarify the cellular origins of circulating circRNAs and uncover context-dependent regulatory roles in metabolic tissues and immune compartments. From a clinical perspective, circRNAs may ultimately contribute to precision medicine frameworks as components of circRNA-based companion diagnostics. Rather than serving as standalone biomarkers, circRNAs could be integrated with clinical parameters, imaging, and other molecular markers to refine risk stratification, predict complication development, or monitor therapeutic responses. Achieving this goal will require standardized assays, robust validation, and clear demonstration of added value over existing diagnostic tools. In summary, while circRNAs represent a promising and rapidly evolving field in T2DM research, significant challenges remain. Addressing methodological limitations, enhancing mechanistic rigor, and pursuing coordinated translational efforts will be critical to fully realize the potential of circRNAs as biomarkers and regulatory targets in metabolic disease. Future progress in this field will also depend on integrating circRNA biology with high-resolution transcriptomic technologies. Although true single-cell circRNA atlases of T2DM islets remain limited, recent large-scale human islet single-cell transcriptomic datasets have already defined robust cell-type-specific alterations across non-diabetic, pre-diabetic, and T2D states. In parallel, a 2025 spatial transcriptomics dataset of human pancreas sections from donors with normal glucose tolerance and T2D has provided an important framework for resolving gene-expression programs in anatomical context (147). Together, these resources create a strong foundation for future circRNA mapping at single-cell and spatial resolution, which will be critical for identifying cell-type-restricted circRNAs, clarifying intercellular communication, and improving mechanistic precision in T2DM islet biology.

## 8 Conclusions

Accumulating evidence indicates that circular RNAs (circRNAs) represent a novel layer of regulatory and diagnostic molecules in type 2 diabetes mellitus (T2DM), linking transcriptomic remodeling to metabolic dysfunction and clinical phenotypes. Through their unique ability to regulate gene expression via competing endogenous RNA (ceRNA) mechanisms, circRNAs are emerging as key players in the complex pathological processes underlying T2DM, including insulin resistance, β-cell dysfunction, inflammation, and vascular complications. The identification of circRNAs as stable, disease-specific biomarkers provides a promising avenue for early diagnosis and stratification of T2DM patients, while their role in metabolic regulation offers mechanistic insights into the disease’s progression. As research progresses, circRNAs are poised to become not only diagnostic tools but also potential therapeutic targets, informing treatment strategies and monitoring responses to interventions. However, challenges remain in terms of standardizing circRNA detection, addressing cohort heterogeneity, and validating mechanistic insights. Moving forward, large-scale, multicenter studies, along with the integration of cutting-edge technologies like single-cell and spatial transcriptomics, will be essential to fully realize the potential of circRNAs in T2DM. Ultimately, circRNA-based companion diagnostics and therapeutic approaches could help pave the way for precision medicine in diabetes care, providing more personalized and effective treatment options for patients.
